# Splenic macrophages as the source of bacteraemia during pneumococcal pneumonia

**DOI:** 10.1016/j.ebiom.2021.103601

**Published:** 2021-10-04

**Authors:** David Carreno, Joseph J Wanford, Zydrune Jasiunaite, Ryan G. Hames, Wen Y Chung, Ashley R. Dennison, Kornelis Straatman, Luisa Martinez-Pomares, Manish Pareek, Carlos J. Orihuela, Marcos I. Restrepo, Wei Shen Lim, Peter W. Andrew, E. Richard Moxon, Marco R Oggioni

**Affiliations:** 1 Department of Genetics and Genome Biology, University of Leicester, Leicester, UK; 2 Department of Hepatobiliary and Pancreatic Surgery, University Hospitals of Leicester, Leicester, UK; 3 Advanced Imaging Facility, University of Leicester, Leicester, UK; 4 School of Life Sciences, University of Nottingham, Nottingham, UK; 5 Department of Respiratory Sciences, Leicester, University of Leicester, UK; 6 Department of Microbiology, University of Alabama at Birmingham, USA; 7 South Texas Veterans Health Care System and University of Texas Health, San Antonio, USA; 8 Department of Respiratory Medicine, Nottingham University Hospitals NHS Trust, Nottingham, UK; 9 Department of Paediatrics, University of Oxford, Oxford, UK; 10 Department of Pharmacy and Biotechnology, University of Bologna, Italy

**Keywords:** Bacteraemia, sepsis, human organ, pathogenesis, pneumonia, therapy

## Abstract

**Background:**

Severe community-acquired pneumococcal pneumonia is commonly associated with bacteraemia. Although it is assumed that the bacteraemia solely derives from pneumococci entering the blood from the lungs it is unknown if other organs are important in the pathogenesis of bacteraemia. Using three models, we tested the relevance of the spleen in pneumonia-associated bacteraemia.

**Methods:**

We used human spleens perfused *ex vivo* to explore permissiveness to bacterial replication, a non-human primate model to check for splenic involvement during pneumonia and a mouse pneumonia-bacteraemia model to demonstrate that splenic involvement correlates with invasive disease.

**Findings:**

Here we present evidence that the spleen is the reservoir of bacteraemia during pneumonia. We found that in the human spleen infected with pneumococci, clusters with increasing number of bacteria were detectable within macrophages. These clusters also were detected in non-human primates. When intranasally infected mice were treated with a non-therapeutic dose of azithromycin, which had no effect on pneumonia but concentrated inside splenic macrophages, bacteria were absent from the spleen and blood and importantly mice had no signs of disease.

**Interpretation:**

We conclude that the bacterial load in the spleen, and not lung, correlates with the occurrence of bacteraemia. This supports the hypothesis that the spleen, and not the lungs, is the major source of bacteria during systemic infection associated with pneumococcal pneumonia; a finding that provides a mechanistic basis for using combination therapies including macrolides in the treatment of severe community-acquired pneumococcal pneumonia.

**Funding:**

Oxford University, Wolfson Foundation, MRC, NIH, NIHR, and MRC and BBSRC studentships supported the work.

**Research in context**

**Evidence before this study**

There is a consensus that moderate and high severity community-acquired pneumonia (CAP) should be treated with a combination of a macrolide and a beta-lactam, but there is not a clear understanding of the mechanisms underpinning this measure. Recently we showed that intracellular replication of pneumococci in CD169+ tissue macrophages precedes invasive pneumococcal disease (IPD), in murine and porcine models, and that this could be prevented by macrolides, raising the hypothesis that an intracellular phase of bacterial replication could precede bacteraemic disease, but there was no evidence that this occurs during pneumonia or in the human spleen.

**Added value of this study**

Using a human spleen *ex vivo* perfusion system, we show that pneumococci are detectable in the splenic macrophages, where they form foci of increasing bacterial numbers over time. In a non-human primate pneumococcal pneumonia model, bacterial foci also formed, and they were still observed after beta-lactam treatment. In a murine model of pneumococcal pneumonia, pneumonia was unaltered histologically and bacteriologically, by treatment with a sub-inhibitory dose of the macrolide, azithromycin. The treatment abolished bacterial counts in spleen and the blood, indicating that it is the bacterial load and functionality of the spleen that determines eventual blood counts and IPD.

**Implications of all the available evidence**

The data provide compelling evidence that the spleen, and not the lungs, is the major source of bacteria giving rise to sustained bacteraemia. We suggest that the evidence provides a mechanistic basis for using combination therapy, that includes macrolides, in treating high severity pneumonia, including pneumococcal pneumonia.

Alt-text: Unlabelled box

## Introduction

Lower respiratory tract infections are the leading cause of mortality from communicable disease [1], with *Streptococcus pneumoniae* causing more deaths than all other lower respiratory tract aetiologies combined [2]. A significant proportion of patients with moderate or high severity community-acquired pneumonia (CAP) develop invasive pneumococcal disease (IPD) characterised by bacteraemia [3,4]. Current treatment guidelines recommend empirical therapy with a beta-lactam for low severity CAP but for patients with moderate and high severity CAP, in whom bacteraemia is likely, a combination of a beta-lactam and a macrolide is recommended [3,5]. Consistent with these recommendations, the addition of macrolide for hospitalised patients with CAP and bacteraemia was found to be associated to reduction of mortality [6,7]. Nevertheless, despite decades of clinical trials and experimental research into CAP and IPD [8], the precise pathogenesis of bacteraemia and the biological rationale for using macrolides is not fully understood.

Recently, we showed that in intravenously infected mice, pneumococcal sepsis is preceded by intracellular replication of the bacteria within splenic macrophages [9]. In the first hours post-infection clearance from the blood was observed, until bacteria were undetectable at eight hours post-infection, but during this eclipse period bacteria could be cultured from the spleen. Confocal microscopy revealed intracellular pneumococci were replicating in CD169+ splenic macrophages. Administration of an anti-CD169 monoclonal antibody completely prevented disease in mice, as did macrolides but not beta-lactams [9]. We demonstrated that in porcine spleens, which have a microarchitecture similar to the human organ, pneumococci replicated in CD169+ macrophages during *ex vivo* perfusion [9,10]. Involvement of CD169+ macrophages provided an explanation for the efficacy of macrolides in preventing late onset sepsis because they, unlike beta-lactams, accumulate within macrophages [11].

Direct extrapolation of our previous findings to human disease management is confounded because the architecture of the human spleen displays significant differences to that of other animals [10,12]. In particular, the human spleen contains three types of tissue macrophages including the red pulp macrophages (CD68+CD163+CD169-) sheath and follicle associated macrophages (CD68+CD163-CD169-), and the peri-arteriolar sheath macrophages (CD68+CD163-CD169++) [10,12]. To address this issue, we obtained evidence from a combination of *ex vivo* human spleen infections, a non-human primate and murine pneumonia models [13], on the role of the spleen in IPD. We also aimed to find a mechanistic explanation for the higher effectiveness of combination therapy that uses macrolides in combination with beta-lactams for moderate and high severity CAP.

Some of the results of these studies have been previously reported in the form of abstracts [14], [15], [16], [17].

## Methods

### Ethics statements

Human spleen perfusions were conducted at the Leicester General Hospital, Leicester, UK. After written informed consent for utilisation of the organ for research, spleens were obtained from patients during elective partial pancreatectomy in the context of pancreatic cancer. The study was approved by the UK Health Research Authority (IRAS219992), the East Midlands-Leicester South Research Ethics Committee (REC18/EM/0057) and has the ClinicalTrials.gov Identifier NCT04620824.

Studies of severe pneumonia in non-human primates (baboons, *Papio cynocephalus*), following intra-bronchial inoculation with *S. pneumoniae*, had been previously performed at the Texas Biomedical Research Institute (TBRI) in San Antonio, Texas [13]. The animal procedures were approved by the Institutional Animal Care and Use Committee (IACUC Number 1443PC6).

Studies with mice were performed at the University of Leicester in accordance with the United Kingdom Home Office licence P7B01C07A and were approved by the University of Leicester Animal Welfare and Ethical Review Body. In accordance with the licence, all mice were scored for signs of disease, culled at pre-determined time points or immediately killed if showing moderate signs of disease.

### Bacterial culture conditions

The classical *Streptococcus pneumoniae* strain D39 (deposited as NCTC 14078) and a more recent clinical isolate TIGR4 (deposited as ATCC BAA-334), respectively serotype 2 and 4, and both not carrying any drug resistance marker [13,18], were used in this work. The serotype 2 strain D39 is the classical pneumococcal strain used for animal infection since the work of Avery in 1944 on the discovery of the DNA as hereditary material [19], and it was the strain used in the discovery of within-macrophage replication of pneumococci [9]. We have added strain TIGR4 in these experiments as type 4 is epidemiologically more relevant, it behaves similarly to D39 in animal models and had been used in the baboon work performed in 2016 of which we analyse stored samples in this work [13,18]. Strains were grown on Brain Heart Infusion medium with 3% v/v defibrinated horse blood (Oxoid, Basingstoke, UK). For infection, pneumococci were cultured to mid-logarithmic phase and aliquots were frozen at -80°C.

### Human spleen perfusion

Normothermic *ex vivo* human spleen perfusion was performed as previously described [10]. Following spleen removal, the splenic artery was cannulated and flushed with Soltran preservative solution (Baxter, Deerfield, IL, USA) and transported to the lab on ice. Organs from patients which had been given antibiotics prior to surgery were excluded. The organs were washed with saline containing 25,000 IU of heparin (LEO Laboratories Limited, Hurley, UK), before connecting to a custom-made, paediatric perfusion circuit (Medtronic, Dublin, Ireland) that included an oxygenator set to 1 L/min, and a 37°C water bath. The organ was perfused with 500 mL of the haemoglobin-based oxygen carrier Hemopure® (HbO2 Therapeutics, Souderton, PA, USA), and a steady infusion of glucose (5% w/v at 30ml/h initially, reduced to 10 or 20 ml/h according to blood gas parameters) and saline supplemented with 500 μg of Flolan (epoprostenol sodium; GlaxoSmithKline, Brentford, UK) and 5,000 IU of heparin (Sigma, St. Louis, MO, USA) to facilitate vasodilation and prevent clotting respectively. Pressure was maintained at 80 Hg/mm, and the flow rate was monitored throughout the perfusion. Once a stable flow rate was reached, pneumococci diluted directly from thawed frozen stocks, were instilled into the circuit via a valve immediately proximal to the splenic artery. Spleen number HSP1 was perfused with 1.5 × 10^8^ CFU and all other spleens with 2 × 10^7^ CFU. Spleens HSP1 and HPS2 were infected with *S. pneumoniae* D39, spleen HSP5 and HSP8 with TIGR4, and spleens HSP6, HSP7, HSP9 and HSP12 with a 1:1 mix of both D39 and TIGR4. Two control spleens (HSP3 and HSP4) were not infected and were used as comparator organs for blood gas analysis. Spleens HSP10 and HSP11 where used for a different project. Hemopure samples and tissue biopsies were taken at predetermined time points for quantitative bacterial culture, or for flash freezing in Optical Cutting Matrix (OCT; CellPath, Newtown, UK) for microscopy analysis. Human tissue samples were excluded from infection experiments if they had been administered antibiotic therapy prior to surgery. Power calculations, statistical analyses, details of immunohistochemistry, the antibodies (Table S2) and microscopy methodologies are detailed in the Supplementary Materials.

### Sample size calculations for murine infections and human spleen perfusions

For calculation of samples sizes for murine infection experiments, we considered a type-1 error rate of 0.05, and a statistical power of 0.8. Using pilot data derived from previous experiments in our group, we anticipated a mean of 10^6^ CFU/g in murine lungs with an anticipated standard deviation of 10^5^. We predicted an effect size of a 20% reduction in CFU/g in azithromycin treated animals. The required sample size using these parameters was 4, but 5 animals were subsequently included per group for animal husbandry purposes.

For human spleen perfusion experiments, we determined samples sizes using pilot data from our published, porcine spleen perfusion model [9,10]. Briefly, for a statistical power of 80% and a significance level of 0.05, and a predicted effect size of a 20% increase or decrease in CFU/G in the tissue, a sample size of 4 organs was calculated. Availability of tissue enabled us to perform infection experiments with a total of 8 spleens in this manuscript.

### Baboon infection model

Fixed spleen sections from baboons (*Papio cynocephalus*) infected with *S. pneumoniae,* available from a previous study, were used for this analysis [13]. In brief, seven adult baboons were inoculated with freshly cultured 10^9^ CFU of *S. pneumoniae* TIGR4, using bronchoscopy [13]. Three baboons were rescued with intravenous ampicillin therapy. Our analysis focused on a comparison of pneumococcal foci sizes within, and association with, different splenic macrophage subsets between non-rescued (n=4) and ampicillin rescued animals (n=3) and, an overview of the experiments and details of individual baboons is provided in supplementary Table S1 [13].

### Murine infection model

Experimental infections complied with the ARRIVE guidelines and the U.K. Animals (Scientific Procedures) Act, 1986. This study utilised 5x adult 6-8-week-old female CD1 mice bred and used at the University of Leicester Pre-clinical Research Facility. Animals were randomly allocated into cages in groups of 5 at 8 weeks of age by staff at the pre-clinical research facility who had no involvement in subsequent experiments using an online, random number generator algorithm. Infections, sample processing and image analysis were performed unblinded. All experimental procedures were performed on animals in the same order throughout to minimise confounding factors associated with different treatment times between animals.

For intranasal infections, mice (n=5 per experimental group, total mice n=50) were lightly anaesthetised with isoflurane, before instilling 10^6^ CFU of pneumococci, diluted directly from thawed frozen stocks, intranasally in 50 μl of BHI [20,21]. For intravenous infections, tail veins of the mice were dilated by placing animals in a 37°C box for 5 minutes, before infection via the lateral tail vein with 10^6^ pneumococci in 100 μl of BHI [18,[22], [23], [24]]. Antibiotics were given 30 min before infection intraperitoneally as a single dose (azithromycin 1.128 µg/g BW; ceftriaxone 0.00624 µg/g BW) and control mice were treated with saline. At pre-determined time points (2, 4, 6, 12, 24 and 48h post challenge), blood was taken from mice by either cardiac puncture under terminal anaesthesia or lateral tail vein bleed and placed into Eppendorf tubes containing 10 µl of 20 mg/ml heparin for CFU counts and in tubes without heparin for serum separation. Azithromycin and ceftriaxone concentrations in serum and homogenised spleen were determined by bioassay performed by IPM (Madrid, Spain), using *Micrococcus luteus* ATCC 9341 as a reference microorganism (details in supplementary materials) [25]. Lungs and spleens were removed post-mortem under aseptic conditions, and either flash frozen in OCT or homogenised for enumeration of bacteria. Our analysis involved the determination of spleen, lung and blood bacterial load differences, alongside signs of disease and lung histology differences, between azithromycin, ceftriaxone and saline-treated groups. In line with the project license, mice would be humanely culled if signs of disease exceed the moderate severity limit, however no animals were prematurely culled or retrospectively excluded from these analyses.

### Immunohistochemistry, microscopy, and image analysis pipelines

Slides of paraffin embedded sections of spleens from baboons with pneumococcal pneumonia were used (Table S1) [13]. The sections were deparaffinised, rehydrated and then subjected to Heat-Induced Epitope Retrieval (HIER) [26]. Briefly, two xylene washes of 3 min each were performed, followed by exposure to a series of decreasing concentrations of ethanol. Later, sections were boiled in sodium citrate at pH 6 (with 0.05% v/v Tween20) for 15 min.

Fresh frozen samples from murine and human spleens were sectioned to 10 μm thickness using a Leica cryostat (CM1950, Leica Microsystems, Wetzlar, Germany). Samples were then fixed in 4% w/v paraformaldehyde for 20 min at room temperature, before being washed three times in PBS. From this stage onwards, formalin-fixed sections subjected to deparaffinisation and antigen retrieval were processed using the same protocol as fresh frozen samples. Samples were permeabilised with 0.1% v/v Triton X-100 in PBS for 10 min and incubated for 30 min in blocking solution (PBS containing 5% v/v donkey or goat serum (Sigma, St. Louis, MO, USA). Sections were incubated for 1 h with primary antibodies diluted in blocking solution, washed three times with PBS, and then incubated for 45 min with secondary antibody solution (Table S2). Samples were then washed three times with PBS and once with H_2_O before adding mounting medium containing DAPI (Thermoscientific ProLong Gold Antifade Mountant, Waltham, MA, USA) and closing the slides with coverslips. All primary and secondary antibodies used in this study are described in Table S2. Haemotoxylin and eosin stains were performed by the University Hospitals of Leicester histology service using standard protocols and analysed blinded.

An Olympus FV1000 confocal laser scanning microscope was used to acquire images using 40x (UPlanFLN 40x/NA=1.3) and 60x (UPlanSAPO 60x/NA=1.35) objectives, and the free software Fiji (v 2.0.0-rc 69/1.52p) [27] for image processing. For 3D visualisation purposes, some images were de-convolved using *Huygens Essential* deconvolution software (v 18.04.1p0 64b) (SVI, Hilversum, Netherlands) and viewed in *Imaris* 3D (v 9.3.1) reconstruction software (Bitplane, Zürich, Switzerland). In order to identify metallophilic CD169+ macrophage distribution in the spleens, sections were scanned using a fully motorised Nikon Eclipse Ti microscope equipped with a Plan Fluor 10X/0.3 objective and an Andor iXonEM+ EMCCD DU 885 camera. Automatic tissue scanning was performed using NIS-Elements software and the images obtained were analysed using ImageJ software.

For scanning microscopy of whole tissue sections, a Vectra Polaris Digital Pathology system (PerkinElmer, Waltham, MA, USA) with fluorescence filter cubes was used for imaging of fluorescently labelled tissue sections, and brightfield acquisition for haematoxylin and eosin stained sections. Both types of sample were imaged using a 40x objective (40x/NA=0.75). Image files were exported in .tiff format and quantitatively analysed using Fiji. For quantitative co-localisation analysis, regions of interest were derived using the Image>Adjust>Threshold function in Fiji using fluorescence signal specific for each macrophage population. These regions of interest were then imposed on the bacterial fluorescence channels for particle analysis.

### Statistical analysis

For statistical analyses Graphpad Prism (v 9.0.0) was used. A test for the normality of the data was performed prior to the statistical tests. For analysis of categorical 2 × 2 contingency tables, a Fisher's exact test was used. For comparison of multiple groups and conditions/time points, appropriate 2-way analysis of variance (ANOVA) tests were performed.

### Role of funding source

The funders had no role in the study design, data collection, data analyses, interpretation, or writing of report.

### Data Sharing Statement

All original microscopy image files are available through the University of Leicester data repository Figshare (https://leicester.figshare.com/) with the doi: https://doi.org/10.25392/leicester.data.12957947. Sharing of human tissue is subject to restrictions by the human tissue act and our trial protocol.

## Results

### Pneumococcal uptake by CD169+ macrophages in the human spleen

**Figure 1:**
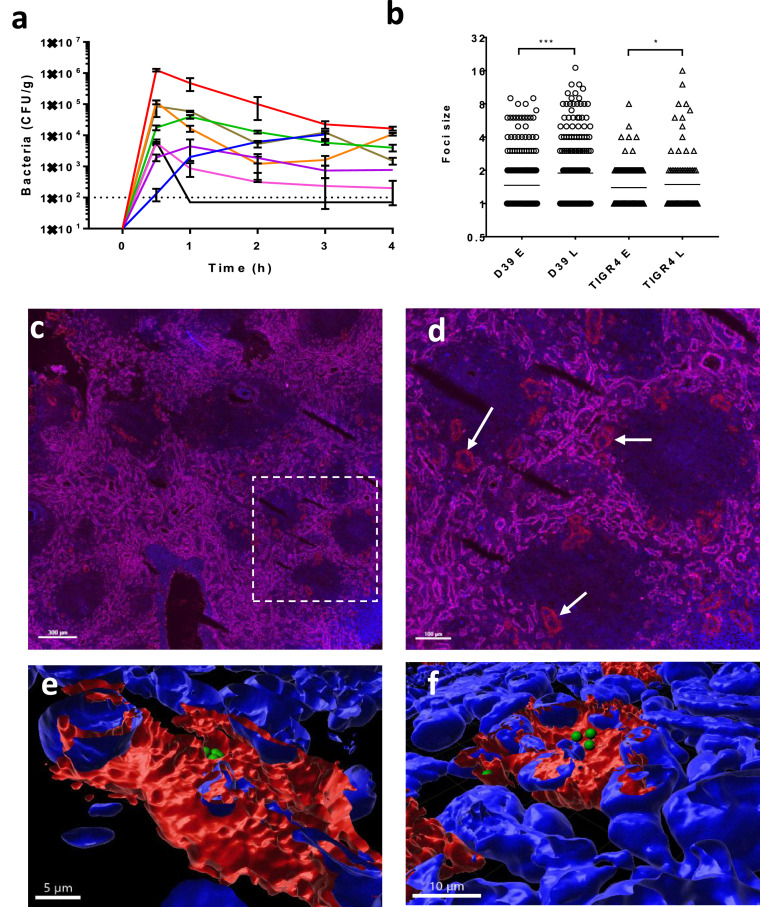
Human spleen *ex vivo* perfusion and infection. Eight human spleens were perfused *ex vivo* with an artificial perfusate and infected with between 2 × 10^7^ and 1.5 × 10^8^ pneumococci. a) Bacterial counts in homogenised spleen biopsies were determined over the time of perfusion (three biopsies from each spleen at each time point). The eight infected perfusion experiments are colour coded HSP1 (red), HSP2 (blue), HSP5 (black), HSP6 (violet), HSP7 (green), HSP8 (pink), HSP9 (orange) and HSP12 (brown). b) The number of bacterial cells in each focus of infection was determined for at least 20 optical fields from at least 3 independent spleen sections in all perfused spleens for early (E; 0.5h) and late (L; 4 or 5h) time points. The breakdown into single spleen details is shown in Fig S1d. Statistical significance was determined by Fischer Exact test (***; p<0.001 * p=0.0324). c) Fluorescent scanning microscopy image of uninfected spleen HSP3 stained for cellular markers (DAPI nuclei, blue; CD206+ sinusoid cells of the red pulp, magenta; CD169+ macrophages, red). Some of the sliced periarteriolar sheaths composed of CD169+ macrophages at the border of the follicle (area devoid of CD206+ sinusoids) are indicated by arrows. The scale bar represents 300µm. d) Zoom of the dashed area of panel c. The scale bar represents 100µm. e-f) Independent representative clusters of pneumococci observed inside different CD169 positive splenic macrophages (red), imaged by confocal microscopy with 3D reconstruction of a Z-stack of microscopy images showing intracellular D39 pneumococci (green) with nuclei stained by DAPI (blue). The scale bars shown represent 5µm (e) and 10µm (f) respectively. All original image files, including a video of both 3-D reconstructions, are available at the Leicester data repository Figshare with the doi: 10.25392/leicester.data.12957947.v1.

To investigate evidence for bacterial replication in human splenic macrophages, after the bacteria had been removed from the blood stream, an *ex vivo* perfusion system was used [10]. Ten human spleens were perfused and eight of them were infected with pneumococci injected into the perfusate. Infection did not alter significantly the flow rate and blood gas parameters (Fig S1b-c). Monitoring bacterial counts in the spleen and perfusate over time (Fig 1a, Fig S1a) showed a common theme of an initial accumulation of bacteria in the organ, indicative of an efficient removal of pneumococci from the perfusate, followed by a gradual decrease until counts eventually stabilised at the later time points (Fig 1a). In the infected spleens the number of foci composed of one/two bacteria decreased over time, accompanied by an increase of foci with larger numbers of bacteria (mono/diplococci for D39 decrease from 87% to 70% and for TIGR4 from 94% to 85%) (Fig 1b, Fig. S1d). In addition to the analysis of pooled samples (Fig 1b), the foci count in three individual organs showed a significant difference in the number of multi-bacterial foci between time points (Fisher Exact Test HSP1 D39 p<0.001, HSP2 D39 p<0.05 and HSP7 D39 p<0.05) (Fig S1d). The absence of bacteria from the perfusate of HSP2 (Fig. S1a), indicates that intracellular replication, and not multi-uptake, was the most likely cause of the increase of multi-bacterial foci.

Scanning microscopy analysis of spleen sections confirmed the presence of CD169-positive periarteriolar sheath macrophages (CD68+CD163-CD169++) on the margin of splenic follicles (Fig 1cd). Confocal microscopy analysis and 3D reconstruction of Z-stack images confirmed that bacterial foci were localised close to eukaryotic nuclei, and within the confines of the macrophage membrane stains, indicating they were intracellular (Fig. 1ef). Image analysis of spleen scans indicated that CD163+ red pulp macrophages outnumbered CD169+ sheath macrophages by about 100-fold with large variations between test areas due to the non-homogeneous micro-anatomy of the spleen (24 samples analysed). Data on infected spleens (n=4) showed that CD169+ macrophages were, after normalisation, at 0.5h and 2h respectively 10.5 and 5.6 times more likely to harbour D39 pneumococci than CD163+ red pulp macrophages (T-Test p<0.05 and ns respectively) and 11.6 and 6.2 times more likely to harbour TIGR4 pneumococci (T-Test ns and p<0.01 respectively). Collectively, these data recapitulate our earlier findings with murine and porcine spleens [9,10]. Moreover, they show that human spleens harbour intracellular foci of *S. pneumoniae* within splenic tissue macrophages following haematogenic infection.

### Clusters of pneumococci in splenic macrophages of baboons with pneumonia

**Figure 2:**
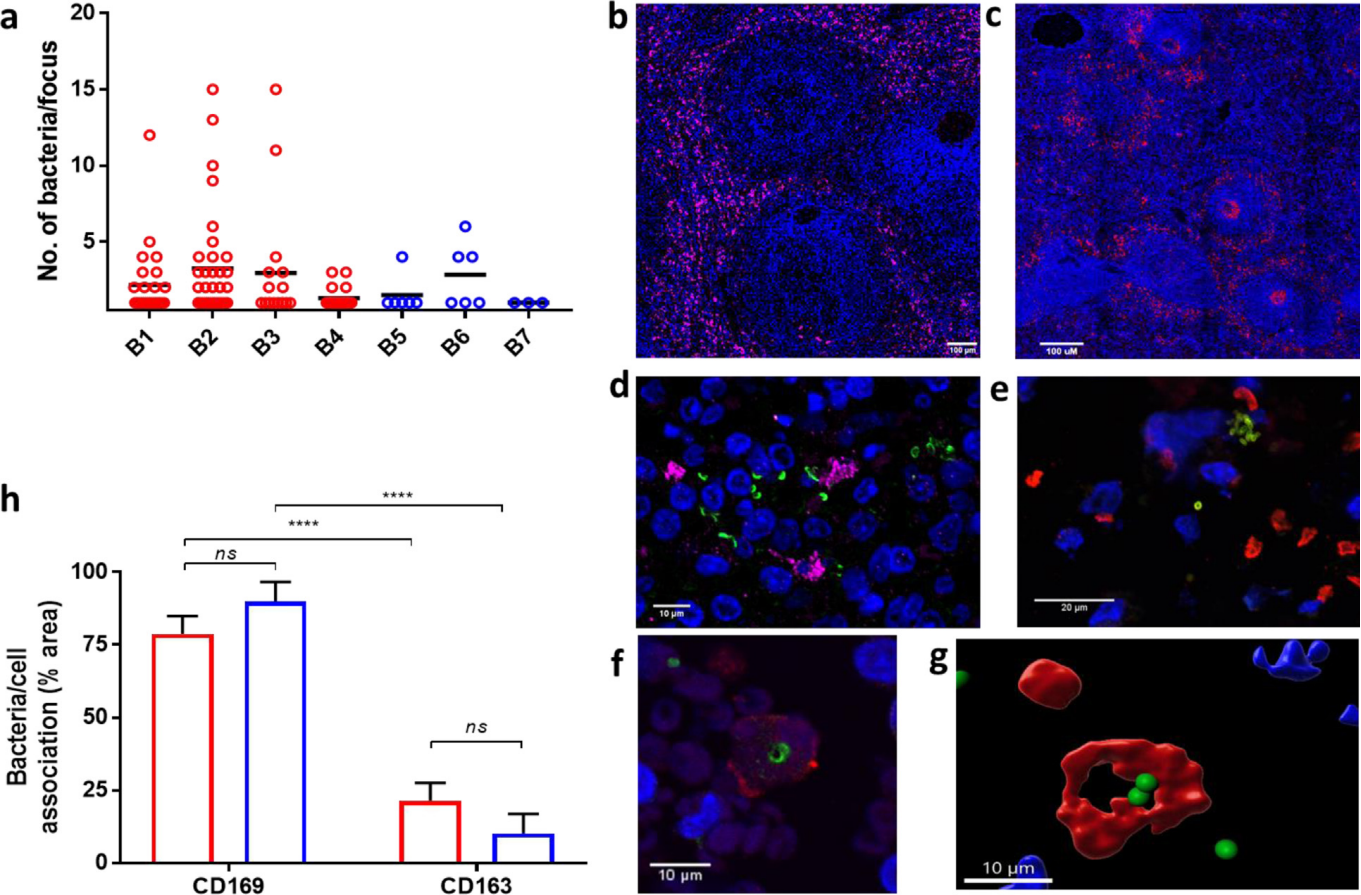
Pneumococci in spleens of baboons during pneumonia. Spleen samples were obtained from stored material of a baboon pneumonia model with *S. pneumoniae* TIGR4 [13]. a) Numbers of bacteria per splenic focus of infection in untreated (red) and ampicillin-treated (blue) baboons (n = 4 and 3 respectively). Bacterial counts were obtained from four random confocal microscopy fields. The number of macrophages with 2 or more bacteria was significantly higher in baboons B1-4 compared to those treated with ampicillin (B5-7), p<0.05. b) A representative image of CD163+ macrophages distribution in the red pulp of a baboon spleen (staining in all panels: bacteria, green; CD169+ macrophages, red; CD163+ red pulp macrophages, magenta; nuclei, blue). The scale bar represents 100µm. c) Perifollicular distribution of CD169+ macrophages. The scale bar represents 100µm. d) Clusters of pneumococcal foci in a non-treated baboon associated with CD163+ red pulp macrophages (magenta). The scale bar represents 10µm. e) Clusters of pneumococci were predominantly observed next to CD169+ peri-arteriolar sheath macrophages in the perifollicular area (CD169, red). The scale bar represents 20µm. f-g) Confocal and 3D reconstruction of the same Z-stack microscopy image showing that pneumococci are associated to CD169+ cells (CD169, red) (the resolution is lower than in Figure 1 due to the necessity for antigen retrieval in deparaffinised samples). The scale bars represent 20µm. h) Distribution of bacteria and their association with tissue macrophages in untreated, bacteraemic (red) and ampicillin-treated baboons (blue) determined using high throughput scanning fluorescence microscopy combined with semi-automated image analysis. Five areas were analysed for all seven baboon spleens. Statistical significance was determined by ANOVA (***, p<0.001; ns, not significant, p>0.05).

To investigate whether there is a link between intracellular pneumococcal foci in the spleen and pneumococcal pneumonia, spleen samples from baboons with experimental pneumonia were analysed [13]. Paraffin-embedded spleen sections from baboons with pneumonia were analysed by scanning and confocal microscopy [13]. Samples were from four infected, but not treated, animals during or shortly after bacteraemic pneumonia (Table S1; animals B1 to 4) and from three infected and ampicillin-treated animals, which, at the time of sampling, had become non-bacteraemic (animals B5 to 7) [13]. Pneumococci were found in the spleen of all seven baboons but the foci in the spleens of untreated baboons (B1-4) had higher numbers of pneumococci compared to the ampicillin-treated baboons (p<0.05) (Fig. 2a). These data clearly showed intracellular pneumococcal foci in the spleen during pneumonia, with intracellular bacteria still detected after treatment, which is consistent with the known inefficient cell penetration of beta-lactams.

To characterise the localisation of the splenic macrophages within the overall micro-architecture of the baboon spleen, whole organ sections were analysed by high-resolution fluorescent scanning microscopy. Images showed a diffuse distribution of CD163+ red pulp macrophages (Fig. 2b), whereas there was a defined localisation of the peri-arteriolar sheath CD169+ macrophages to the peri-follicular area (Fig. 2c). Staining demonstrated that pneumococci associated both with the red pulp CD163+ macrophages (Fig. 2d) and the CD169+ macrophages (Fig. 2e). Quantification of bacteria-macrophage co-localisation in the spleen sections showed that regardless of antibiotic treatment, pneumococci prevalently associated with the splenic CD169+ macrophages during pneumonia (Fig. 2h). Using 3D reconstruction of confocal microscopy Z-stacks, both extracellular pneumococci and intracellular pneumococci within the tissue macrophages, were observed (Fig. 2f-g).

### The spleen, not the lung, is the source of sustained pneumococcal bacteraemia in a mouse model of pneumonia

**Figure 3:**
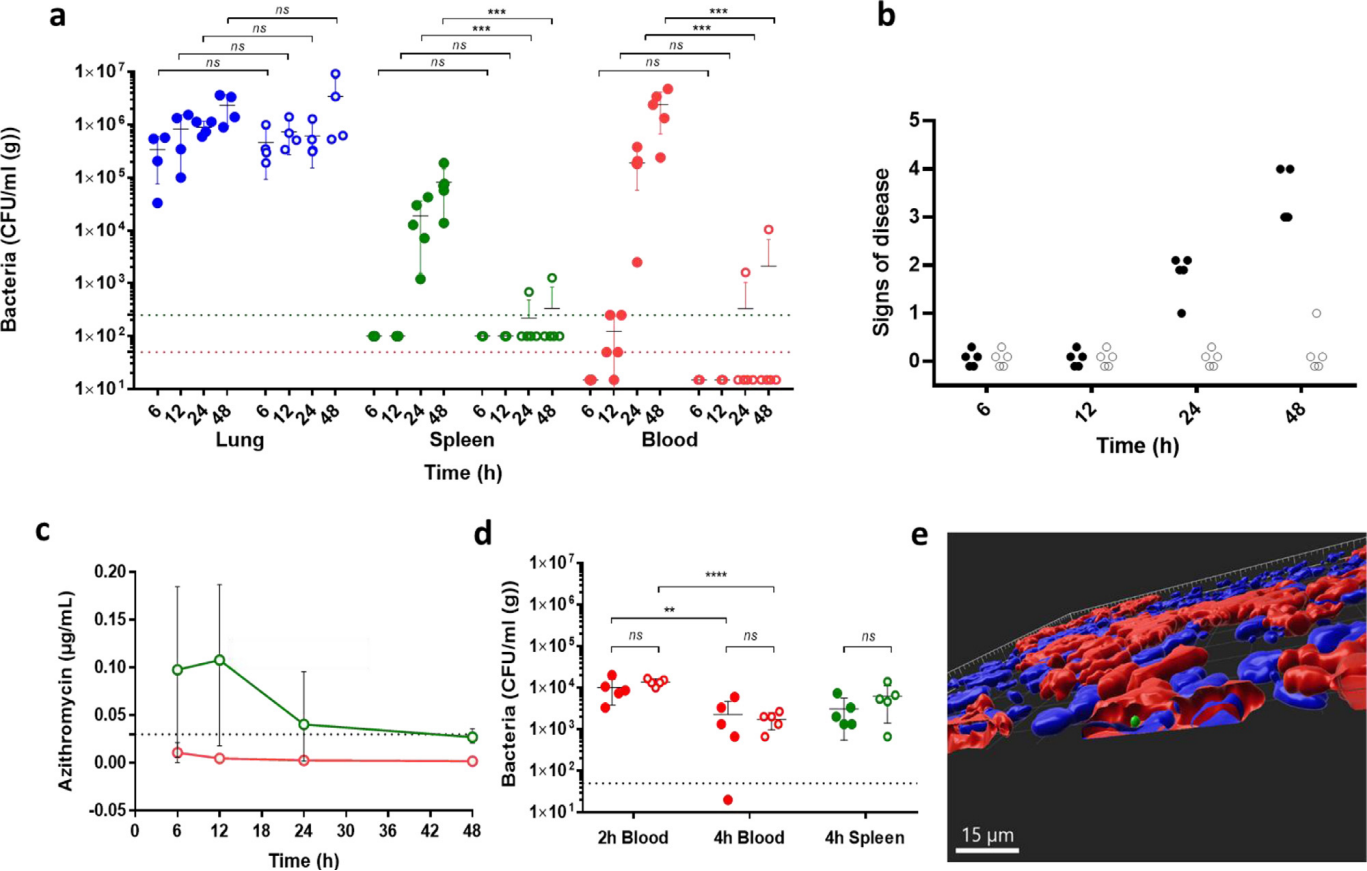
Pneumococci after intranasal challenge of mice and treatment with a low dose of azithromycin. a) Animals (n = 5) were intranasally challenged with 1 × 10^6^ CFU of D39 pneumococci and after 30 min treated intraperitoneally with either a sub-inhibitory 1.128 μg/g BW azithromycin (open circle) or saline (closed circle). The antibiotic dose was chosen to be approximately 10x below the MIC in the serum. Mice were sampled at 6, 12, 24 or 48 hours after infection and bacterial counts were determined for lungs (blue), spleen (green) and blood (red). The limit of detection is shown as dotted line for the spleen and lung (green) and blood (red) samples. b) Signs of disease in mice were assessed and scored as 0 Normal, 1 Hunched, 2 Pilo-erection, 3 Loss of activity, 4 Lethargic, which was the severity limit of the experiment (filled circles: saline, open circles: azithromycin). c) Concentrations of azithromycin were determined by a bioassay of five homogenised spleens (green) and serum (red) of azithromycin-treated mice (values in Table S3). d) Effect of azithromycin treatment (same dose as panel a) on numbers of bacteria in the blood (red) and the spleen (green) after a separate intravenous challenge control experiment (n = 5; open circle azithromycin; closed circle saline). Statistical significance was determined by ANOVA (**, p<0.01; ***, p<0.001; ****, p<0.0001; ns not significant, p>0.05). e) 3D reconstruction of confocal image of CD169+ macrophages in the spleen at 24 h of a saline-treated mouse (CD169, red; bacteria, green; nuclei, blue). The scale bar represents 15µm.

To further investigate if intracellular replication of bacteria within the spleen plays a role in the development of pneumococcal bacteraemia, we used a mouse model of pneumonia and bacteraemia, which followed intranasal infection. To selectively inhibit intracellular organisms, mice were treated with a single dose of azithromycin, a drug with a long half-life that is known to concentrate up to 40-fold within macrophages [28]. The dose of azithromycin was chosen to give a peak serum concentration 10-fold lower than the MIC (azithromycin MIC 0.031 mg/L TIGR4; 0.016 mg/L D39) [29,30], and thus non-inhibitory for blood-borne bacteria. However, at the chosen dose sufficient azithromycin would accumulate intracellularly to achieve clearance of intra-, but not extra-cellular pneumococci. For comparison with a drug that did not accumulate intracellularly, some experiments included ceftriaxone, also given intraperitoneally at the dose to achieve, as for the macrolide, a serum concentration ten times below the MIC (ceftriaxone MIC 0.0312 µg/mL for both strains). To confirm that the azithromycin dose yielded sub-inhibitory concentrations of drug in the serum, but inhibitory concentrations in the tissue, serum samples and organ homogenates from mice at each time were analysed by a bioassay. At all time points, azithromycin serum concentrations were below the MIC (TIGR4 and D39 azithromycin MIC; 0.0312 µg/mL), but above the MIC in the spleen (Fig. 3c). Notably the mice with the lowest tissue concentration of azithromycin at 24 and 48 h, showed low, but positive, spleen and blood counts (Table S3). Ceftriaxone concentrations were below the MIC in both sample sets (Table S4).

In azithromycin-treated and saline-treated mice, bacterial counts in the lungs, over 48 h after infection, were similar (p>0.05) at each time point (Fig. 3a). Bacterial counts in the spleen and blood of sham-treated mice were detectable by 24 h post-infection and were higher by 48 h post-infection (Fig. 3a). Mice not given azithromycin had visible signs of disease at 24 and 48 h post-infection, whereas mice treated with azithromycin did not (Fig. 3b). Furthermore, mice given azithromycin had undetectable or very low numbers of bacteria in the blood at 48 h, when the experiment ended (p<0.001 compared with control), despite having bacterial lung titres indistinguishable from those of the control (p>0.05). Strikingly, mice dosed with azithromycin had significantly lower spleen bacterial numbers compared with saline-treated mice (p<0.001), which correlated with absence of bacteraemia (Fig. 3a). In contrast, the numbers of bacteria in the organs of the mice treated with a sub-inhibitory dose of ceftriaxone was similar to those in the PBS-treated controls (p>0.05) (Fig. S2), in agreement with the lack of intracellular accumulation of this drug (Table S4). Microscopy analysis of spleen sections from the intranasally infected mice revealed foci of pneumococci in association with CD169+ macrophages in the sham-treated group, whereas there were no foci in the azithromycin-treated group (Fig. S3c-d). 3D reconstruction of Z-stacks from the spleens of the sham-treated mice showed that bacteria were present within the confines of the CD169 membrane staining, indicating they were intracellular (Fig. 3e). In these animals, the bacteraemia correlated with greater localisation of bacteria within the splenic red pulp (Fig. S3a). In contrast, the spleens of azithromycin-treated mice showed a normal, non-septic, histology without any increased cellularity in the red pulp and a clearer demarcation in the marginal zone (Fig. S3b).

### Low-dose azithromycin does not alter histology or bacterial distribution in the lungs

**Figure 4:**
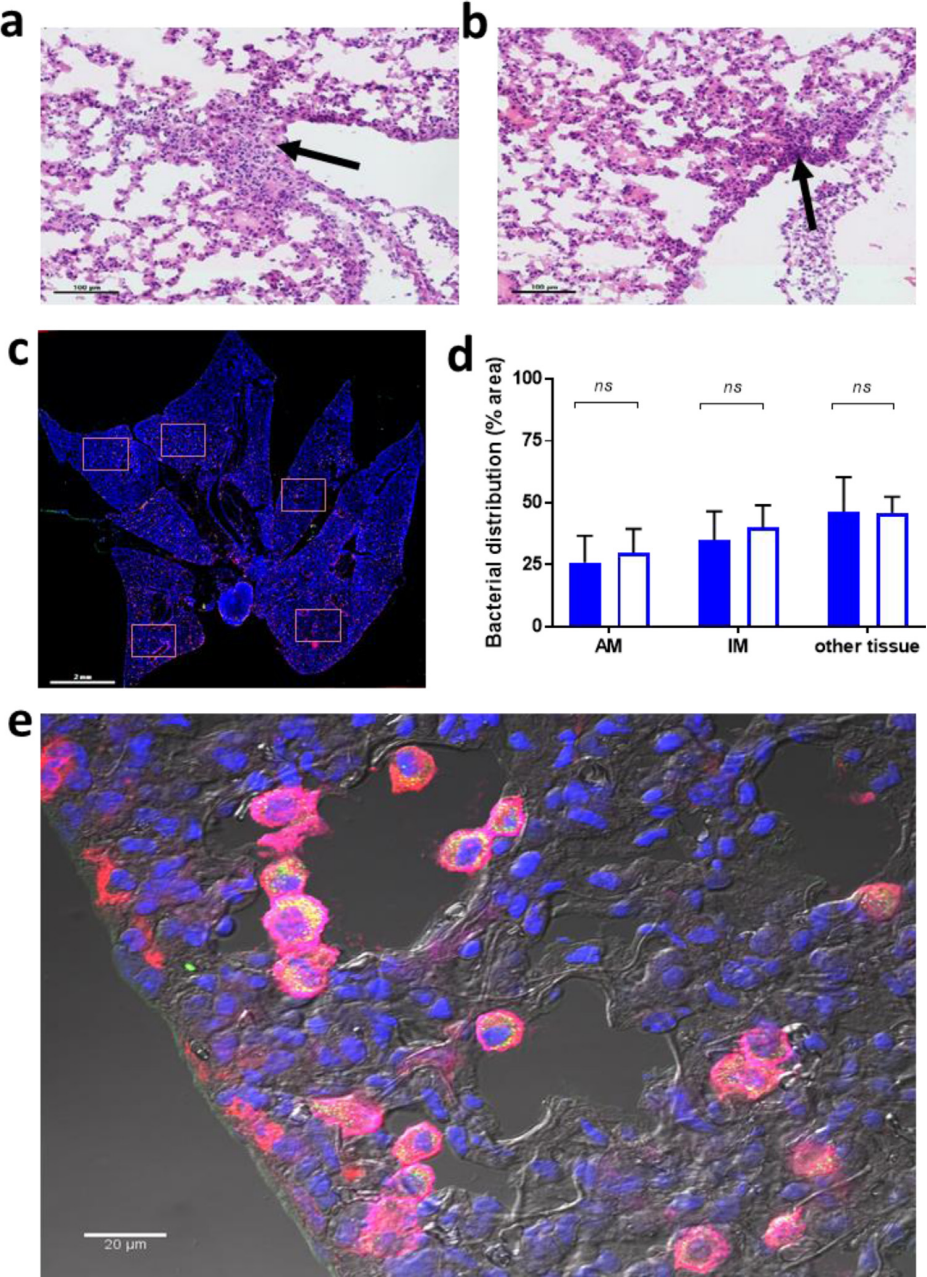
Bacterial distribution and histology of the lungs after intranasal challenge in mice treated with a subinhibitory dose of azithromycin. Two groups of animals (n = 5) were intranasally infected with 1 × 10^6^ CFU and, after 30 min, either treated intraperitoneally with low-dose azithromycin or saline. The animals were culled at 48 h and lungs were collected to analyse pneumococcal distribution and histology of the lungs. Haematoxylin and eosin staining, analysed blinded, showed no differences between control (a) and (b) azithromycin-treated mice, both groups showed the beginning of pneumonia with signs of inflammation in the subpleural area, the edge of the lungs and mesothelial cells. Increase in cellularity is indicated by an arrow. The scale bars represent 100µm. c) Whole lung tissue sections visualised by scanning microscopy. For each sample, 5 random areas were used to analyse bacterial distribution (squares). The scale bar represents 2mm. d) Distribution of bacteria in alveolar macrophages (AM, CD11c+ CD169+) interstitial macrophages (IM CD11c- CD169+) and in unstained tissue (other tissue) in azithromycin-treated (open bar) or saline-treated mice (filled bar) (five independent fields analysed for each of the 5 treatment and 5 control mice). Statistical significance was determined by ANOVA (*; p<0.05, ns; not significant). e) Representative phase contrast image of bacterial (in yellow/green) distribution within AM (magenta) and IM (red) in the lung using a 60x confocal objective. Arrows point to some of examples of AM containing bacteria and bacterial debris. The scale bar represents 20µm.

To confirm that treatment with azithromycin did not alter the histopathological presentation of pneumonia, which could have influenced translocation of organisms to the blood, sections were prepared of lungs collected from mice 48 h post-infection. Haematoxylin and eosin staining indicated that lung pathology was indistinguishable between saline-treated (Fig. 4a) and azithromycin-treated mice (Fig. 4b), with both displaying histological features of bronchopneumonia, including inflammatory infiltrates in the subpleural area and in close proximity to the mesothelial cells. Sections from saline- and azithromycin-treated mice were then stained with antibodies against CD11c and CD169, to allow differentiation of alveolar macrophages (CD169+ CD11c+) and interstitial macrophages (CD169+ CD11c-) [31] (Fig. 4c). It was observed that in both groups of mice there was a similar association of bacteria with alveolar and interstitial macrophages (Fig. 4d-e), indicating there were no off-target effects of azithromycin in the lung.

### Azithromycin does not enhance microbicidal activity of splenic macrophages

Macrolides have been reported to have immunomodulatory effects on macrophages [32]. Therefore, to test if the reduction in splenic bacteria in azithromycin-treated mice could due to enhancement of the microbicidal activity of splenic macrophages, one group of mice was treated with azithromycin and another with saline, 30 min prior to intravenous infection [18]. We observed that comparable (p>0.05) clearance of bacteria from the blood occurred between 2 and 4 h post-infection in both groups of mice with comparable spleen counts (Fig 3d), confirming that the dose of azithromycin neither changed clearance of bacteria from the blood nor the killing of bacteria in the spleen.

## Discussion

Using *ex vivo* perfusion of the human spleen system, together with models of pneumococcal pneumonia in a non-human primate and in the mouse, we have found compelling evidence that the spleen, and not the lungs, is the major source of bacteria giving rise to sustained bacteraemic disease. The evidence also provides a mechanistic explanation for the observed benefit of combination beta-lactam-macrolide antibiotic therapy over beta-lactam monotherapy in the management of moderate and high severity CAP, including pneumococcal pneumonia.

For many years, lower respiratory infections have been the most common cause of mortality from communicable disease and *S. pneumoniae* is the most frequent etiological agent [1,2]. There is a consensus in treatment guidelines worldwide that empirical treatment for moderate and high severity CAP should be with combined beta-lactam and macrolide antibiotics [3,5]. This is supported by a meta-analysis of studies on the clinical outcome of CAP and bacteraemia showing reduced mortality with macrolide-containing regimens [6], but the mechanism underpinning this decreased mortality is not known. In hospitalised adults with proven pneumococcal pneumonia, treatment with a combination of a beta-lactam and macrolide antibiotic has also been found to be associated with lower mortality compared to beta-lactams alone [33,34]. Possible explanations for the added beneficial effect of macrolides include; 1) a broader spectrum of antibiotic activity encompassing penicillin-resistant organisms, although clinically relevant resistance to beta-lactams is infrequent [35]; 2) concurrent anti-inflammatory or immunomodulatory effects reducing proinflammatory cytokine responses [32]; and 3) interference with pneumococcal virulence factors such as pneumolysin [36]. Our findings suggest a novel and previously unrecognised explanation.

Sustained bacteraemia and sepsis following pneumococcal pneumonia is generally assumed to result from continuous seeding of bacteria from the lung into the bloodstream. We proposed a different hypothesis in which bacteria enter the blood from the lung, are filtered by the spleen and only following intracellular replication within splenic CD169+ macrophages do they initiate sustained bacteraemia. This hypothesis does not challenge the primacy of splenic macrophages in clearing capsulated bacteria and that loss of the spleen predisposes to overwhelming sepsis [37,38]. Rather, it emphasises the complexity afforded by the differing roles of distinct populations of splenic tissue macrophages [39]. The validity of our hypothesis was testable because sub-inhibitory concentrations of azithromycin, at serum concentrations that would not prevent seeding of bacteria from the lung to the blood, accumulates within cells and reach bactericidal levels. If sepsis depended on seeding from the lungs, then at these sub-inhibitory doses sepsis would ensue unabated. But, if a stage of intracellular replication was critical to sustaining bacteraemia, then the intracellular accumulation of azithromycin would kill the bacteria and prevent sepsis under circumstances where lung bacterial counts were unchanged. The prevention of sepsis in a mouse model of pneumococcal pneumonia, using these sub-inhibitory concentrations of azithromycin in the lung, provides compelling evidence in support of our hypothesis.

Extrapolation of the observations in mice to humans is constrained by the very different splenic architecture of murine spleens compared to that of humans [12]. Our studies with the non-human primate spleen, which has a similar structure to humans and with human spleens themselves [40], both show that splenic macrophages are relevant to human pneumococcal disease. These findings extend the relevance of the mouse model to human infection.

In spleens of pneumonic baboons, we observed clusters of pneumococci associated with splenic macrophages. The presence of these foci, even following treatment with ampicillin (which does not effectively penetrate macrophages), provides evidence for an extravascular, reservoir for pneumococci where they may be protected from innate immune effectors. The observation of bacterial foci in the spleens of ampicillin-treated baboons indicate that this intracellular niche may allow pneumococci to survive monotherapy with beta-lactams. This is in line with recent data showing persistence of pneumococci within alveolar macrophages of asymptomatic subjects for days after experimental carriage and amoxicillin treatment [41], which suggest that sequestered bacteria are not eradicated by treatment doses of beta-lactams, which is of relevance to management of human infection.

Next, we sought evidence that in human spleens CD169+ macrophages are permissive for pneumococcal replication. For this, we established a perfusion system in which human organs could be maintained and subsequently infected *ex vivo* [10,42]. Microbiological and microscopic analysis of these experimental infections provide the first evidence that human spleens contain replication-permissive macrophages, a novel concept in the pathogenesis of pneumococcal disease in humans [9,10]. Previous reports with human macrophages (primarily alveolar macrophages or monocyte-derived macrophages) have shown effective killing of pneumococcus, which is further enhanced by opsonisation (a confounding factor that our model eliminates) [43,44], but not intracellular replication of the pneumococcus in human cells. Macrophages are phenotypically highly heterogeneous, and many subsets, e.g. alveolar macrophages and inflammatory monocyte-derived macrophages have potent antibacterial killing mechanisms [45]. While pneumococcal foci were more likely in human CD169+ sheath-associated macrophages, they were more frequent in red-pulp macrophages but, as previously observed in both mice and pigs [9,10], neutrophils in the red pulp are expected to clear those potential foci of infection. In contrast, splenic CD169+ macrophages, seem to be primarily equipped for antigen presentation and lack a strong killing activity [46,47]. This conclusion is consistent with ability of pneumococci to replicate within them, but it does raise the question of the mechanism by which bacteria enter these cells that are ill-equipped to deal with them. The observation in three models that the splenic macrophages are a reservoir for bacteraemic infection is consistent with the observed superior efficacy of macrolides in the mouse and in the treatment of human pneumococcal pneumonia where bacteraemia plays a major role in the severity of the disease [3,48]. Macrolides concentrate within eukaryotic cells at a far greater concentration that other antibiotics, which corresponds with comparatively lower clinically achievable serum concentrations [11,49].

Our findings provide a new mechanistic basis for current recommendations for the use of a combination of beta-lactam and macrolide antibiotics in the empirical management of moderate and high severity CAP, particularly in bacteraemic disease where *S pneumoniae* is the predominant pathogen [3,5,6]. They also provide a strong rationale for a clinical trial of combination therapy versus targeted beta-lactam monotherapy in microbiologically confirmed severe pneumococcal pneumonia.

## Caveats and Limitations

We used three distinct experimental systems, each of which has inherent technical and physiological limitations. In the human spleen perfusion model, given the limited availability of organ samples, we elected to use a low inoculum of pneumococci. Our rationale was to simulate natural infection and avoid the potential criticism of bacterial overload. This resulted in very few bacteria per biopsy section, especially for the TIGR4 strain. It should be noted that we pooled the data from experiments that used variable conditions for perfusing the artificial blood substitute, a judgement that could be legitimately questioned. The baboon sample analysis was conducted on 5-year-old paraffin embedded spleen samples that were available from a previous experiment whose experimental aims were unrelated to the present study. Potential limitations include: the sample storage medium and unexplained interference of IHC staining of macrophages with our CD169 antibody in the paraffin embedded sections that resulted in poor visualisation of the marker, but the results provide additional support for our hypothesis using a pneumonia model in non-human primates. The murine infection addresses our hypothesis the most directly but is limited by the treatment parameters. Given all these limitations, it is the nevertheless the sum of all three approaches, not any one data set, that provides substantial preclinical evidence to justify further investigation in clinical trials.

## Contributors

All authors read and approved the final version of the manuscript. DC, JJW, ZJ and RGH performed experiments and wrote the manuscript, WYC and ARD performed surgery and perfusion, KS supervised microscopy, LMP provided guidance on immunology, MP and WSL provided guidance on clinical relevance, CJO and MIR contributed the baboon experiments, PWA supervised the mouse experiments, and PWA, ERM and MRO did the project planning, contributed funding and verified the underlying data.

## Declaration of Competing Interest

MRO reports grants from MRC MR/M003078/1, grants from BBSRC BB/S507052/1 , grants from MRC IMPACT Doctoral Training program, during the conduct of the study; grants from GSK co-funding of CASE studentship BBSRC BB/S507052/1, outside the submitted work; and consultancy for Pinsent Masons LLP 2020-21 on work unrelated to this manuscript. MRO had a BactiVac Network grant BVNCP3-05 2019-20 with GSK on topics unrelated to this manuscript. ZJ reports grants from BBSRC CASE GSK studentship BB/S507052/1, outside the submitted work. MP reports grants and personal fees from Gilead Sciences, personal fees from QIAGEN, grants from Sanofi, outside the submitted work. WSL has received core support from the NIHR Nottingham Biomedical Research Centre, and his institution has received unrestricted investigator-initiated research funding from Pfizer for an unrelated multicentre cohort study in which WSL is the Chief Investigator. The other authors have nothing to disclose.

## Appendix Supplementary materials

Image, video 1

Image, application 2

Image, application 3

Image, application 4
