# Supplementary material for: Splenic macrophages as the source of bacteraemia during pneumococcal pneumonia
Source: eBioMedicine. 2021 Oct 4;72:103601. doi: 10.1016/j.ebiom.2021.103601 (PMC8498229; doi:10.1016/j.ebiom.2021.103601)
Supplement: Supplementary file 3 [file mmc3.docx]

**Supplementary Data**

**Splenic macrophages as the source of bacteraemia during pneumococcal pneumonia**

David Carreno, Joseph J Wanford, Zydrune Jasiunaite, Ryan G. Hames, Wen Y Chung, Ashley R. Dennison, Kornelis Straatman, Luisa Martinez-Pomares, Manish Pareek, Carlos J. Orihuela, Marcos I. Restrepo, Wei Shen Lim, Peter W. Andrew, E. Richard Moxon, Marco R Oggioni

- Table S1: Overview of the experimental details of individual pneumonic baboons.
- Table S2: Antibodies.
- Table S3. Azithromycin concentration and bacterial counts in spleen and serum samples.
- Table S4. Ceftriaxone concentration and bacterial counts in spleen and serum samples.
- Figure S1: Human spleen perfusion data.
- Figure S2: Pneumococci counts in the blood, spleen and lungs after intranasal challenge in mice treated with sub-inhibitory azithromycin and ceftriaxone.
- Figure S3: Histology of the spleens after 48h of intranasal challenge with TIGR4 in mice treated with azithromycin sub-MIC.

**Table S1**. Overview of the experimental details of individual pneumonic baboons

| **Animal** | **Day after challenge** | | | | | | | | | | | | | |
| --- | --- | --- | --- | --- | --- | --- | --- | --- | --- | --- | --- | --- | --- | --- |
|  | **1** | **2** | **3** | **4** | **5** | **6** | **7** | **8** | **9** | **10** | **11** | **12** | **13** | **14** |
| **1** |  | B | B | BE |  |  |  |  |  |  |  |  |  |  |
| **2** |  | B | B | B | B | B | BE |  |  |  |  |  |  |  |
| **3** |  | B |  |  |  |  |  |  | E |  |  |  |  |  |
| **4** | B | B | B | B | B | B | E |  |  |  |  |  |  |  |
| **5** | B | B | B | B | B | A | A | A | A | A | A | A | A | AE |
| **6** | B | B | B | B | BA | A | A | AE |  |  |  |  |  |  |
| **7** | B | B | B |  | A | A | A | A | A | AE |  |  |  |  |

B bacteraemia, A ampicillin treatment, E endpoint (spleen sampling) [13].

**Table S2.** Antibodies.

| **Antibody** | **Abbreviation** | **Specificity** | **Conjugate** | **Catalogue** | **Supplier** |
| --- | --- | --- | --- | --- | --- |
| Anti-human CD169 | α-CD169h | CD169 | None | MA1-16891 | Thermoscientific |
| Polyclonal Anti-human Siglec-1/CD 169 | α-CD169ph | CD169 | None | AF5197 | R&D Systems |
|  |  |  |  |  |  |
| Anti-human CD163 (OTI2G12) | α-CD163 | CD163 | None | ab156769 | Abcam |
| Anti-human CD206 | α-CD206 | CD206 | None | MCA2155 | Bio-Rad |
| Anti-mouse CD11c (N418) | α-CD11c | Alveolar macrophage | None | 14-0114-82 | Thermoscientific |
| Anti-mouse CD169 (3D6.112) | CD169 | Siglec-1 | None | MA5-16508 | Thermoscientific |
| Anti-pneumococcal Omni serum | Omni | Bacteria | None | 2481 | Statens Serum Institut |
| Anti-pneumococcal type 4 capsule | α-type4 | Bacteria | None | 16747 | Statens Serum Institut |
| Goat anti-Rabbit IgG (H+L) | AF488 | Secondary Ab | Alexa Fluor® 488 | A32731 | Thermoscientific |
| Goat anti-Rat IgG (H+L) | AF568 | Secondary Ab | Alexa Fluor® 568 | A-11077 | Thermoscientific |
| Donkey anti-sheep IgG (H+L) | AF568d | Secondary Ab | Alexa Fluor® 568 | A21099 | Thermoscientific |
| Donkey anti-mouse IgG (H+L) | AF647d | Secondary Ab | Alexa Fluor® 647 | A-31571 | Thermoscientific |
| Goat anti-Armenian hamster 647 IgG (H+L) | AF647g | Secondary Ab | Alexa Fluor® 647 | ab173004 | Abcam |
|  |  |  |  |  |  |

**Table S3**. Azithromycin concentration and bacterial counts in spleen and serum samples

|  |  |  | **Spleen** | | |  | **Serum** | |
| --- | --- | --- | --- | --- | --- | --- | --- | --- |
| **Sample** |  |  | **µg/mL** | **µg/g** | **CFU/g** |  | **µg/mL** | **CFU/ml** |
| **6h** | **1*** |  | 0.011 | 0.073 | <250 |  | <0.002 | <50 |
|  | **2** |  | 0.185 | 1.360 | <250 |  | 0.027 | <50 |
|  | **3** |  | 0.104 | 0.693 | <250 |  | 0.010 | <50 |
|  | **4** |  | 0.183 | 1.028 | <250 |  | 0.014 | <50 |
|  | **5** |  | 0.006 | 0.032 | <250 |  | <0.002 | <50 |
|  |  |  |  |  |  |  |  |  |
| **12h** | **1** |  | 0.108 | 0.684 | <250 |  | 0.006 | <50 |
|  | **2** |  | 0.187 | 1.230 | <250 |  | 0.012 | <50 |
|  | **3** |  | 0.112 | 0.566 | <250 |  | <0.002 | <50 |
|  | **4** |  | 0.115 | 0.599 | <250 |  | 0.004 | <50 |
|  | **5** |  | 0.018 | 0.159 | <250 |  | <0.002 | <50 |
|  |  |  |  |  |  |  |  |  |
| **24h** | **1** |  | 0.076 | 0.524 | <250 |  | <0.002 | <50 |
|  | **2** |  | 0.010 | 0.059 | <250 |  | <0.002 | <50 |
|  | **3** |  | 0.096 | 0.549 | <250 |  | 0.006 | <50 |
|  | **4** |  | <0.002 | 0.013 | 691 |  | <0.002 | 1600 |
|  | **5** |  | 0.019 | 0.142 | <250 |  | <0.002 | <50 |
|  |  |  |  |  |  |  |  |  |
| **48h** | **1** |  | 0.021 | 0.136 | 1266 |  | 0.002 | 10500 |
|  | **2** |  | 0.031 | 0.173 | <250 |  | <0.002 | <50 |
|  | **3** |  | 0.024 | 0.136 | <250 |  | <0.002 | <50 |
|  | **4** |  | 0.036 | 0.218 | <250 |  | <0.002 | <50 |
|  | **5** |  | 0.024 | 0.158 | <250 |  | <0.002 | <50 |

* Samples were collected from five mice at each time point and analysed by a bioassay by IPM (Madrid). CFU values of each of the animals in spleen and blood. The limit of detection in the spleen was 250 CFU/g and in the blood 50 CFU/ml.

**Table S4**. Ceftriaxone concentration and bacterial counts in spleen and serum samples.

|  |  |  | **Spleen** | | |  | **Serum** | |
| --- | --- | --- | --- | --- | --- | --- | --- | --- |
| **Sample** |  |  | **µg/mL** | **µg/g** | **CFU/g** |  | **µg/mL** | **CFU/ml** |
| **48 h** | **1*** |  | ≤0,004 | ≤0.028 | 5.9 x 10^4^ |  | 0,023 | 3.0 x 10^3^ |
|  | **2** |  | ≤0,004 | ≤0.025 | 1.1 x 10^6^ |  | ≤0,004 | 6.5 x 10^6^ |
|  | **3** |  | 0,024 | 0.031 | 1.9 x 10^4^ |  | ≤0,004 | 1.0 x 10^3^ |
|  | **4** |  | ≤0,004 | ≤0.031 | 1.3 x 10^6^ |  | ≤0,004 | 4.5 x 10 ^6^ |
|  | **5** |  | ≤0,004 | ≤0.035 | 5.8 x 10^3^ |  | 0,023 | 3.5 x 10^3^ |

* Samples of five mice were collected at each time point and analysed by Bioassay (IPM, Madrid). CFU values of each of the animals in spleen and blood. Limit of detection (LOD) in the spleen - 250 CFU, LOD in the Blood – 50 CFU.

**
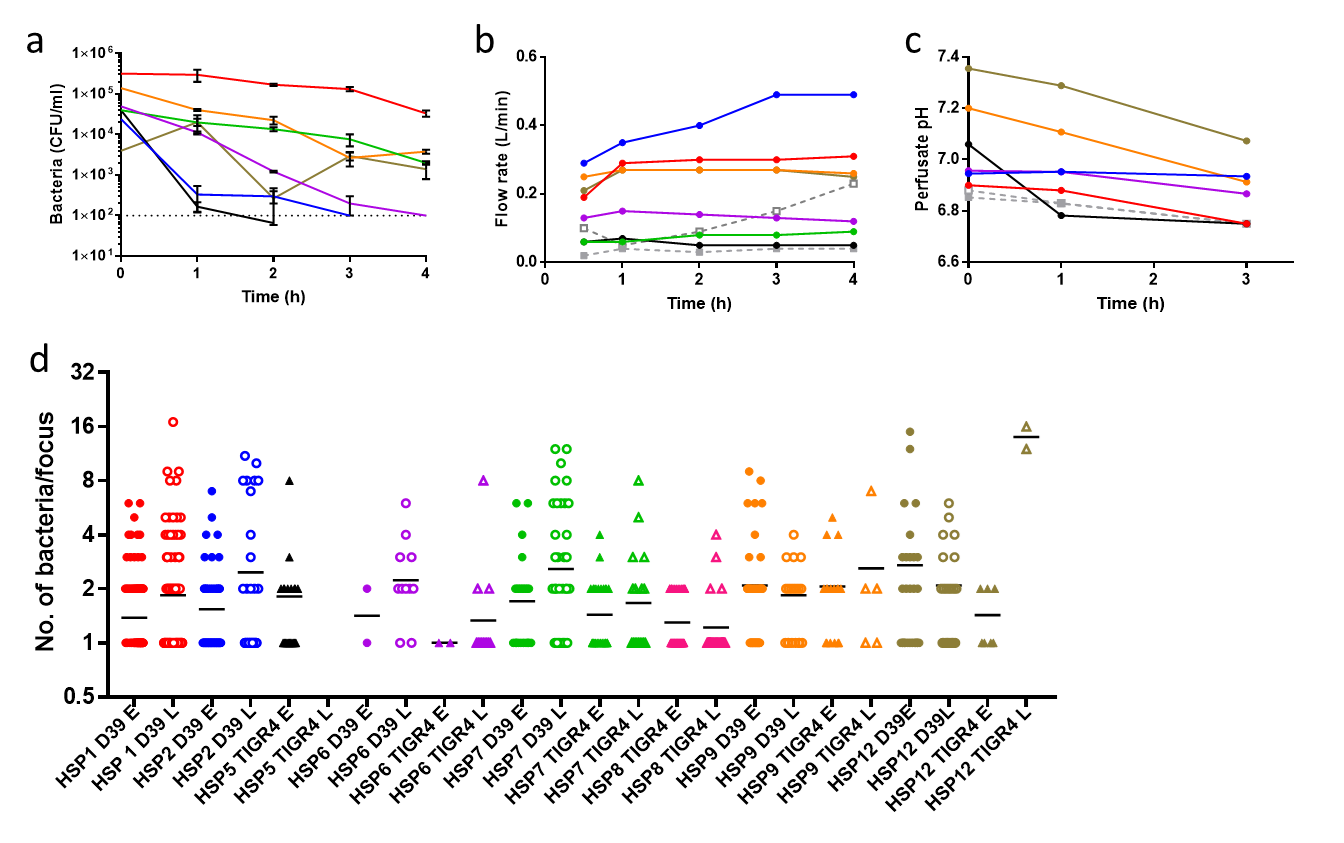
**

**Figure S1: Human spleen perfusion.** Eight human spleens were perfused *ex vivo* and infected with 2x10^7^ pneumococci (except spleen HSP1 with 1.5 x 10^8^ CFU). a). Bacterial counts were determined in the perfusate over time after infection of the perfusion fluid (three samples at each time point). The eight infected spleens are colour coded: HSP1 (red), HSP2 (blue), HSP5 (black), HSP6 (violet), HSP7 (green), HSP8 (pink), HSP9 (orange) and HSP12 (brown). b) The flow rate of the perfusion as reported on the perfusion controller (colour codes are as in S1A, with the addition of the two control spleens, HSP3 (grey, dashed open square) and HSP4 (grey, dashed, filled square). c) pH of the perfusate over time. d) The detail for each spleen of the number of bacteria in foci counted by microscopy in the earliest (E; 30 min; filled symbols) or final biopsy sample (L; 3, 4 or 5 h; open symbols) (colour codes are as in S1A) (20 optical fields from 3 independent spleen sections). Data for bacterial counts in foci for both *S. pneumoniae* D39 (circles) and TIGR4 (triangles) are shown.

**
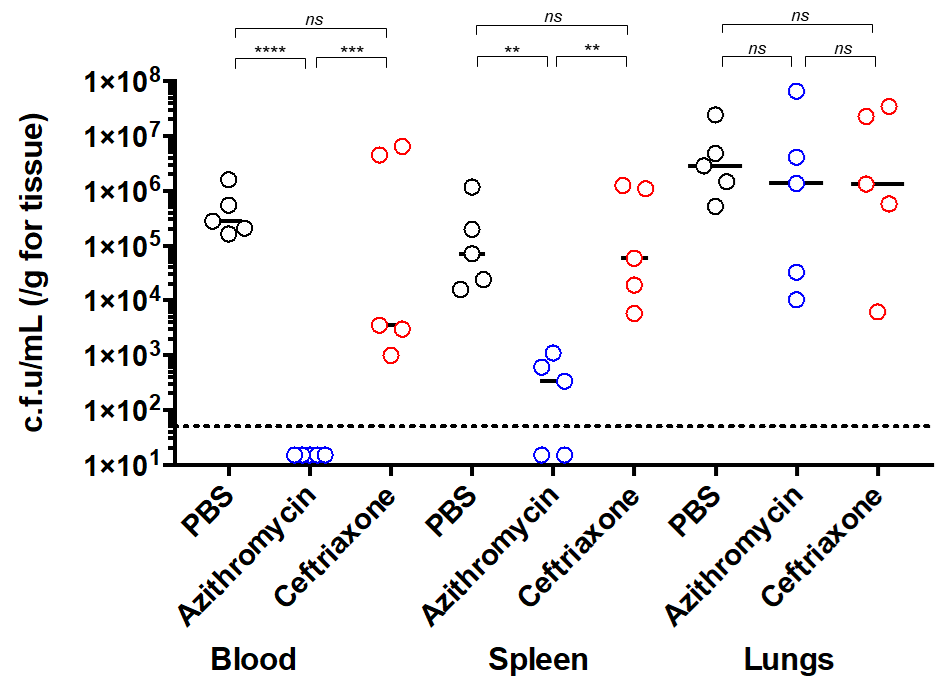
**

**Figure S2. Pneumococcal counts in the blood, spleen and lungs after treatment of intranasally infected mice with sub-inhibitory azithromycin or ceftriaxone.** Three groups of animals (n = 5) were intranasally challenged with 5 x 10^5^ CFU of *S. pneumoniae*. After 30 min, azithromycin (blue), ceftriaxone (red) (the serum concentration of each antibiotic was targeted to be 10x below the MIC) or saline (black) were given intraperitoneally. Bacterial counts in blood, spleen and lung were determined at 48 h. Statistical significance were determined by ANOVA (*; p<0.05, ns; not significant, p>0.05).


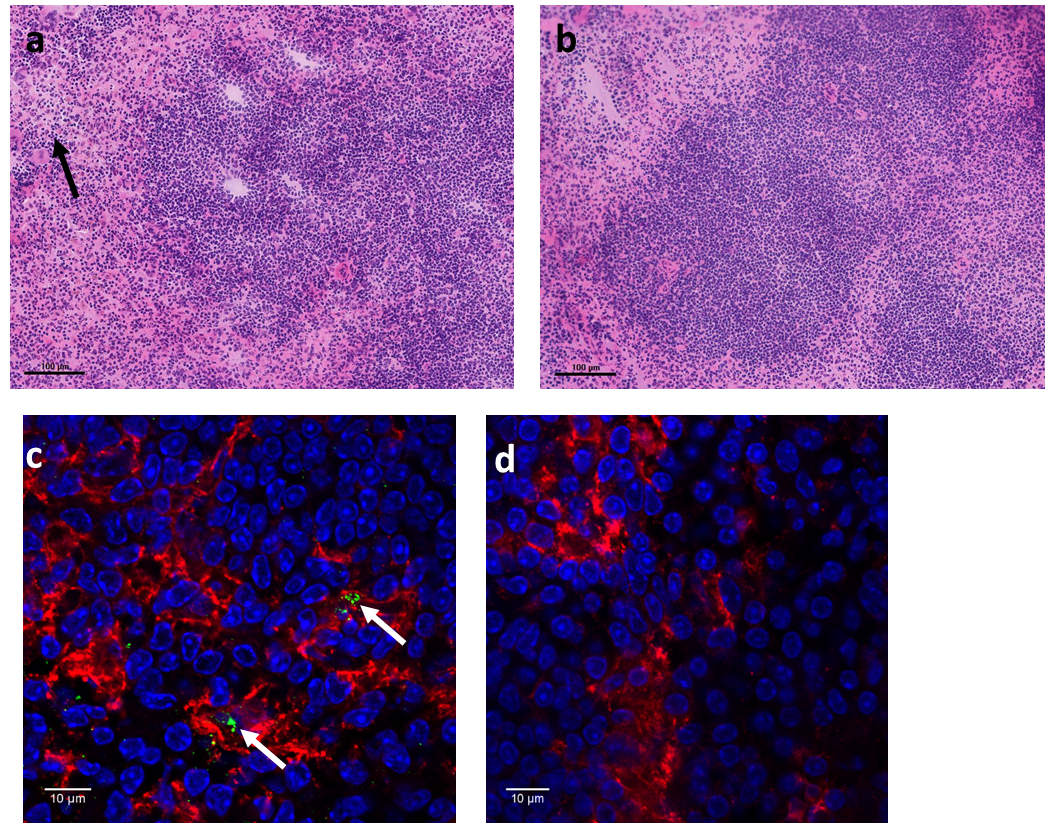


**Figure S3. Histology of murine spleens** **48h after intranasal infection with *S. pneumoniae* and treatment with sub-inhibitory azithromycin.** Two groups of animals were intranasally challenged with 1 x 10^6^ CFU of *S. pneumoniae*. After 30 min, azithromycin estimated to be approximately 10x below the MIC or PBS (control) was intraperitoneally injected. a) 48 h after infection, the spleens of bacteraemic mice of the saline-treated group show an increase in cellularity within the red pulp (arrow) and a clear demarcation of the marginal zone. The scale bar represents 100µm. b) At 48 h after infection, spleens of non-bacteraemic azithromycin-treated mice showed less cellularity in the red pulp and a less clear demarcation of the marginal zone. The scale bar represents 100µm. c-d) Confocal images of spleen sections of saline-treated show presence of bacteria (arrows) (c) while no bacteria are detected in samples from azithromycin-treated mice (d) (CD169, red; bacteria, green; nuclei, blue). The scale bars represent 10µm.
